# The influence of cavity design on the mechanical behavior of endo-crown restorations: an ex-vivo study

**DOI:** 10.1186/s12903-025-06586-y

**Published:** 2025-07-19

**Authors:** Mohamed Gomaa Altamimi, Omaima El Mahallawi, Monika Lukomska-Szymanska, Mohammed Turky

**Affiliations:** 1 Department of Fixed Prosthodontics, Faculty of Dentistry, Minia University, Minia, Egypt; 2 Ministry of Health and Population, Kafr El Sheikh, Desouk, Egypt; 3 Department of Fixed Prosthodontics, Faculty of Dentistry, Cairo University, Cairo, Egypt; 4 Department of General Dentistry, Medical University of Lodz, Lodz, Poland; 5 Department of Endodontics, Faculty of Dentistry, Minia University, Minia, Egypt; 6 Department of Endodontics, Faculty of Dentistry, Sphinx University, Assiut, Egypt

**Keywords:** Cavity design, Endo-crown, Fracture resistance, Mechanical performance, Root canal-treated teeth

## Abstract

**Background:**

Root canal-treated teeth often exhibit significant structural loss, necessitating effective and conservative restorative techniques. The present study aimed to assess the effect of cavity design on the fracture resistance of endo-crown restorations.

**Materials and methods:**

Thirty human mandibular first molars were carefully chosen based on a sample size estimation. All teeth underwent root canal treatment and were allocated into three groups (*n* = 10 each) according to the cavity design; conventional cavity design (group 1), conventional cavity design with a root canal extension (group 2), and conventional cavity design with a buccal groove (group 3). Following cementation of the endo-crown restorations and submission to thermo-mechanical fatigue, each group was subjected to fracture resistance testing and failure mode analysis. A one-way ANOVA and Tukey’s post hoc tests were conducted to compare the mean fracture load values, while the Chi-square test was used to compare failure modes among the study groups. The significance was set at 5% with a confidence interval (CI) of 95%.

**Results:**

The conventional design demonstrated the highest fracture resistance, significantly outperforming the other designs (*P* < 0.0001). The root canal extension design showed the lowest resistance, while the buccal groove design exhibited intermediate results (*P* < 0.0001). Failure mode analysis revealed distinct patterns, indicating that the conventional design with root canal extension fractured under the lowest loads, yielded the most favorable outcomes (*P* < 0.0001).

**Conclusions:**

The choice of cavity design significantly impacts the mechanical performance of endo-crown restorations. Endo-crown restorations exhibited the highest fracture resistance in the conventional cavity design. Moreover, the design extending into a buccal groove can be preferred over the extension into the root canals.

**Clinical significance:**

The findings support using conventional designs to boost the longevity and effectiveness of restorations in root canal-treated teeth. When additional extensions are required, a buccal groove extension is suggested rather than an extension into the root canal. This study contributes valuable insights for clinicians in selecting optimal restorative strategies for root canal-treated teeth.

**Clinical trial number:**

Non-applicable. Conducting the current experiment was limited to the approval by the local Research Ethics Committee at the Faculty of Dentistry, Minia University, Egypt (committee no. 94, registration no. 705, dated 28 February 2023).

## Introduction

Root canal-treated teeth (RCTT) with substantial tissue loss pose a clinically challenging situation, with long-term survival relying on choosing the most appropriate restoration along with maintaining the remaining tooth structure [1–3]. RCTT are prone to shatter because their dentin has less flexibility, a decreased moisture level, and considerable tooth loss due to caries removal, traumatic injuries, access cavity preparation, and restoration replacement [4, 5].

Effective restoration of RCTT requires a comprehensive understanding of their biomechanical and physical characteristics, anatomy, and a solid grasp of periodontal, endodontic, restorative, and occlusal principles [6]. It is crucial to accurately assess the strength of the remaining tooth structure by drawing a conclusion about which section of the tooth structure plays the most significant role in maintaining stiffness as well as fracture resistance [7, 8]. In the presence of a suitable ferrule, mutilated RCTT have traditionally been restored with a post and core followed by a full coverage restoration [9–11]. Nevertheless, the preparation of post space might increase the possibility of root weakening, perforation, or fracture [12]. Furthermore, a crown lengthening surgery carries surgical risks, including increased sensitivity of neighboring teeth, harm to the supporting tooth, and an increase in healing time and expense [13, 14].

In recent times, adhesive strategies have led to a paradigm shift toward post-less restorations and the utilization of the pulp chamber as an extension, integrating the crown and core as a single unit, or monobloc. This approach forms the basis of the endo-crown modality [15, 16].

Endo-crown restorations refer to single-piece restorations that replace a part of the tooth crown while projecting into the pulp chamber of posterior teeth to provide resistance as well as retention forms [17]. Those minimally invasive bonded restorations were introduced by Pissis between 1989 and 1992 [18]. They are recommended in case of extensive tooth loss, particularly when there are limited interproximal and occlusal spaces. In such situations, the inadequate ceramic thickness makes rehabilitation using post and crown unfeasible [19].

In addition, endo-crown restorations have emerged as a viable option for extensively damaged RCTT with insufficient retention form, curved and/or short roots, short clinical crowns, and calcified roots [17, 20, 21]. This might be attributed to the macro retention provided by the axial walls of the pulp chamber combined with the use of adhesive luting cement [17]. Moreover, endo-crown restorations have been suggested as a treatment modality for restoring RCTT in patients with specific occlusal considerations due to the superior fracture resistance of endo-crown restorations when compared to the hybrid post/core/crown restorations [22]. These restorations offer several advantages, including esthetics, simplicity in fabrication, practicality, conservativeness, cost-effectiveness, less time-consuming, and promoting favorable stress distribution [5, 23].

Despite all these benefits, shallow cavities present a significant confrontation to the retention and stability of endo-crowns under both functional and para-functional loads [24]. However, this issue can be mitigated by incorporating additional means during cavity preparation to improve the restoration’s resistance and retention. Therefore, the success of endo-crown restorations relies partly on the design of the prepared cavity and subsequent restoration design. Modifying these designs can impact the stress distribution as well as the failure patterns of endo-crown restorations [17]. The conventional cavity design employed for endo-crown restorations is the most widely adopted design due to its straightforward nature and its effectiveness in preserving a significant amount of the remaining tooth structure [25]. However, this design presents challenges related to retention and resistance, particularly in instances involving teeth characterized by thin and/or weakened axial walls, diminished cavity depths associated with shallow pulp chambers, or those that experience heightened occlusal forces. These factors collectively increase the risk of catastrophic failures [24–26].

Considering these limitations, various alternative approaches have been proposed and researched. For instance, designs that incorporate extensions into the root canals have been investigated to enhance mechanical interlocking and improve stress distribution [27]. Nevertheless, a definitive consensus on the most effective cavity design ensuring the long-term clinical success of endo-crown restorations remains elusive. Furthermore, the introduction of axial grooves as an additional technique aimed at bolstering both retention and resistance in endo-crown cavity preparations without jeopardizing critical tooth structures such as peri-cervical dentin has yet to be thoroughly explored, highlighting a significant gap in the current literature. Hence, the present research aimed to evaluate the effect of the cavity design - the conventional cavity design, the root canal extension design, and the axial groove extension design - on the biomechanical behavior of endo-crown restorations. The null hypothesis tested is that there was no difference in mechanical performance between the endo-crown restorations in different cavity designs.

## Materials and methods

### Ethical considerations

The present study was conducted in strict adherence to the ethical principles set forth in the Declaration of Helsinki, which seeks to ensure the safety and rights of participants involved in medical research. Approval for the study was granted by the Research Ethics Committee at the Faculty of Dentistry, Minia University, Egypt, under committee number 94 and registration number 705, on February 28, 2023. To ensure compliance with all relevant ethical guidelines and regulations, the research methodologies employed were thoroughly reviewed and implemented with precision. Furthermore, written informed consent was obtained from all participants prior to their involvement in the study, ensuring that they were fully aware of the research objectives, procedures, potential risks, and benefits. This process prioritized transparency and respect for the autonomy of individuals participating in the study.

### Sample size calculation

Based on a previous study [28], the sample size was calculated to detect statistical significance among groups using the analysis of variance test. The means were estimated at 308.26, with a significance level (alpha) set at 0.05 (two-tailed), a confidence interval of 95%, an effect size of 1.3, and a beta (β) level of 0.2. Consequently, a sample size of 10 in each group provides an 80% power for this experiment. This report was generated using GraphPad StatMate version 2.00 for Windows (GraphPad Software, San Diego, CA, USA).

### Sample selection according to strict criteria

Thirty intact recently extracted human mandibular first molar teeth with fully developed two roots were selected. Teeth extracted with minimal trauma for periodontal reasons were collected from healthy patients 20–30 years old from the outpatient clinic, Faculty of Dentistry, Minia University, Minia, Egypt. To mitigate the risk of bias due to anatomical variations among the selected samples, all teeth were submitted to 3D imaging (Papaya 3D plus, Genoray, Gyeonggi-do, Korea) in order to accurately match the teeth morphology, dimensions, and pulp space configuration and volume. All chosen teeth exhibited comparable volumes of pulp chambers, equivalence in terms of root/root canal curvature angles as measured by Schneider’s approach [29], radii (less than 5 mm), and the same canal configurations with Vertucci’s type IV in the mesial root and type I in the distal root [30]. Moreover, the dimensions of the teeth were standardized by measuring the mesiodistal and buccolingual width using a digital caliper with an accuracy of 0.01 mm. The mesiodistal dimensions were 11 mm ± 0.5 mm, while the buccolingual dimensions were 8 mm ± 0.5 mm. In an effort to standardize tooth length, the entire occlusal surface was reduced to adjust the full length to 20 mm.

Carious teeth and those with pre-existing restorations, root canal filling, calcification, and resorptive lesions were discarded and replaced with teeth, strictly following the inclusion criteria. Additionally, teeth were checked under a dental operating microscope (DOM) at a magnification of 20× (Magna Labomed, Labo America Inc., 920 Auburn Court Fremont, CA, USA) to exclude evidence of cracks and fractures.

Selected teeth underwent ultrasonic cleaning to eliminate remaining hard or soft deposits followed by disinfection in 2.5% sodium hypochlorite solution (NaOCl; El Nasr Company for Intermediate Chemicals, Giza, Egypt) for 30 min. Lastly, in compliance with the technical specification ISO/TS 11,405 established in 2003 in the field of Dental materials, teeth were stored for a maximum of one month prior to testing in a 0.1% thymol solution (Formula e Acao, São Paulo, SP, Brazil) [31].

### Experiment conditions

Controlling variables such as humidity and temperature is essential for ensuring the reproducibility and reliability of laboratory studies. Hence, all samples were tested on the same day in a humidity and temperature-controlled laboratory room. This room was equipped with heating systems, and air conditioning was used to uphold a constant temperature of 23 °C ± 1 °C, as suggested in ISO 4049 for testing dental materials. Humidifiers and dehumidifiers were employed to create stable humidity levels.

### Teeth mounting

A self-curing resin was poured into molds (25 mm in height and 30 mm in diameter). Each tooth, with a stretch film of 0.3 mm in thickness covering its root, was placed in acrylic resin 2 mm below the cementoenamel junction (CEJ) to replicate the bone level. Teeth were positioned using a Dental Surveyor (Ney Dental Surveyor, Anaheim, CA, USA) to ensure proper centering and alignment parallel to the long axis of the tooth. Following the setting of acrylic resin, the teeth with the stretch film were removed and reinserted after injecting a light body silicone (Elite HD+; Zhermack SpA, Badia Polesine, Italy) into the root cavity to simulate the periodontal ligament. Any excess impression material was eliminated using a #12 blade (Swann Morton, Sheffield, England).

### Root canal procedures

All teeth received identical root canal procedures, including access cavity preparation, root canal instrumentation, irrigation protocol, and root canal filling materials and technique, and were conducted by a single experienced endodontist (M.T.) in a single session under a magnification of 16× using DOM. A round diamond bur (Komet 801 − 014; Komet Den Komet Dental, Braseler GmbH & Co. KG, Lemgo, Germany) was used to penetrate the pulp chamber and remove its entire roof. To ensure a straight-line path to the canal orifices, the Endo-Z bur (Dentsply-Maillefer, Ballaigues, Switzerland) was then utilized to refine the cavity walls, creating slightly divergent walls. The burs used for preparing the access cavity were attached to a high-speed handpiece with water cooling. Following the traditional access preparation, which was standardized using a digital caliper with an accuracy of 0.01 mm, root canal patency was assessed using a stainless-steel K-file ISO size 10 (Kiyohara Industrial Park, Utsunomiya, Tochigi, Japan). Teeth with initial apical diameters of 0.15 mm and 0.25 mm (equivalent to file sizes 15 and 25) for the mesial and distal root canals, respectively, were selected. The working length was determined visually by inserting a size 10 K-file until its tip was visible at the apical foramen, then subtracting 1 mm from the obtained measurement. Root canals were instrumented up to apical sizes 0.35 mm and 0.40 mm (equivalent to file sizes 35 and 40) for the mesial and distal root canals, respectively using Nickel-titanum (NiTi) rotary file system (Hyflex CM; Coltene Whaledent, Cuyahoga Falls, OH, USA) mounted on a controlled torque endodontic motor (NSK, Tokyo, Japan) and used in a continuous rotation motion after adjusting the torque and speed according to the manufacturer’s recommendations. Between mechanical files, irrigation of root canals was carried out using 2 mL of 2.5% NaOCl for 20 s. The final irrigation protocol was accomplished by utilizing 10 mL of 2.5% NaOCl for 2 min succeeded by 10 mL of 17% ethylenediaminetetraacetic acid (EDTA; Pulpdent Corp, Watertown, MA, USA) for 2 min with saline irrigation in the same volumes and for the same periods in between to avoid sequelae of combining both irrigants and as a final flush to combat the persistent EDTA-softening of the canal walls [32].

The root canals were dried with the matched paper points (Coltene Whaledent, Cuyahoga Falls, OH, USA) and filled using a single cone technique with the matched gutta-percha cones (Coltene Whaledent, Cuyahoga Falls, OH, USA) and a bioceramic sealer (META BIOMED CO.LTD, 270, Osongsaengmy1-ro, Osong-eupHeungdeok-gu, Cheongju, Chungcheongbuk-do, Korea). Excess gutta-percha was removed up to 1 mm below the canal orifices to enhance the endo-crown adaptation, and the pulp chamber was cleansed from any excess sealer using 70% alcohol to not negatively affect the adhesive bond of the endo-crown restoration [33]. In addition, cavities were rinsed with an anti-oxidant agent (10% sodium ascorbate for 2 min) followed by water rinsing to inhibit the residual effect of NaOCl (oxygen by-products) on adhesive polymerization [30, 34]. By the completion of root canal procedures, teeth were examined under a magnification of 20× using the DOM for the incidence of cracks or fractures before fabricating endo-crown restorations and testing.

### Experimental grouping

The sample distribution involved stratification, where the specimens were pair-matched based on their dimensions and anatomy. Teeth that had comparable dimensions and anatomical characteristics were divided into three different groups according to the cavity design. Specifically, each set of three anatomically and dimensionally matched teeth was assigned to separate groups to minimize the risk of bias arising from anatomical variations among the selected samples.

**Fig. 1:**
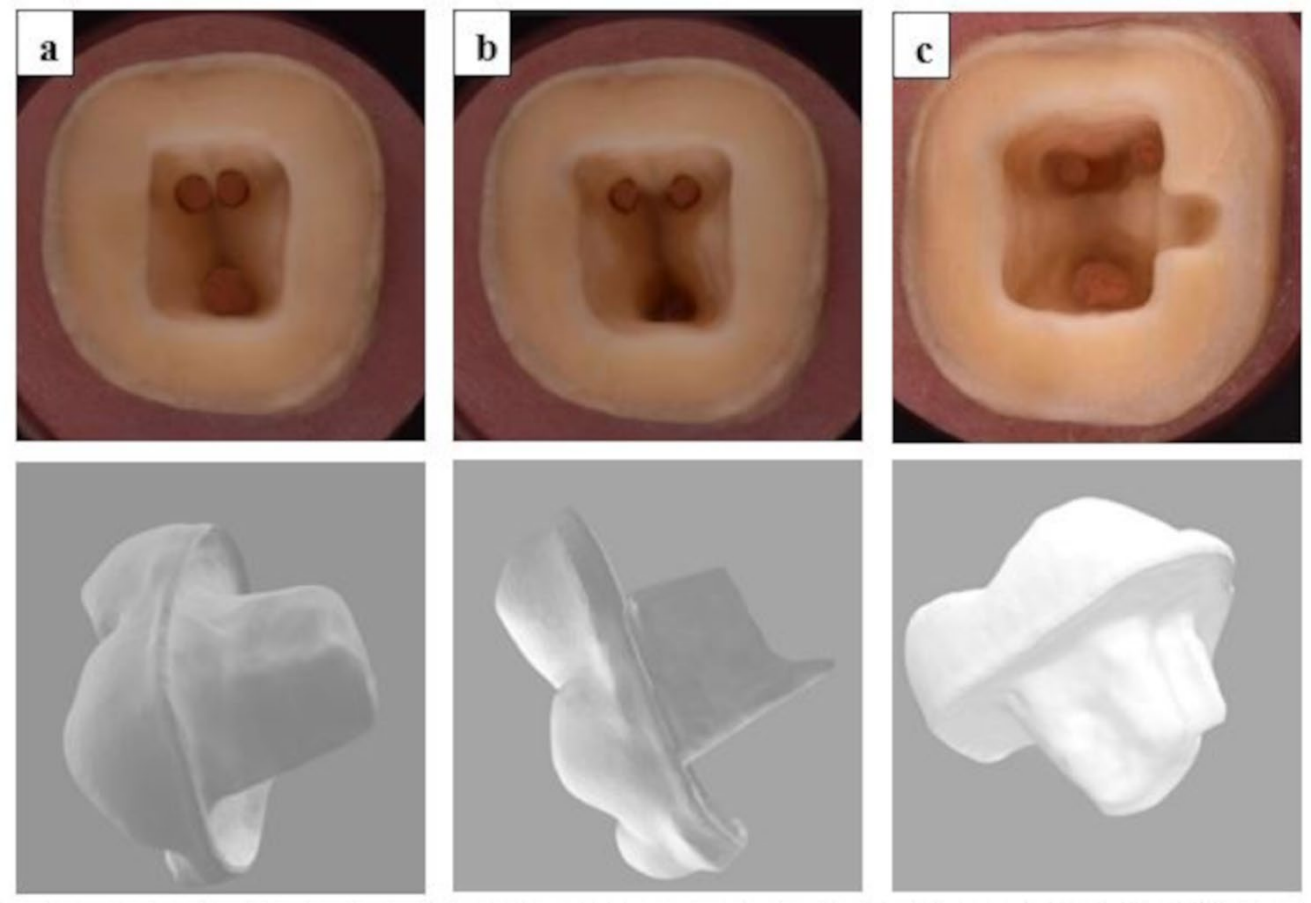
Representative images for the different cavities and respective endo-crown designs. **a**: The conventional cavity design, **b**: The conventional cavity design with a root canal extension, **c**: The conventional cavity design with a buccal groove extension

The three groups were as follows:

- Conventional design (Group 1):
  Prior to occlusal reduction, a 2 mm guided groove was prepared on the occlusal surface using a tapered flat-ended diamond bur (Komet 845KR; Komet Den Komet Dental, Braseler GmbH & Co. KG, Lemgo, Germany) followed by using a wheel diamond bur (Komet 8856; Komet Den Komet Dental, Braseler GmbH & Co. KG, Lemgo, Germany) to ensure uniform 2 mm reduction of the occlusal table, resulting in a 90^o^ circumferential band of 1–2 mm enamel margins (butt-joint) in order to improve bonding and establish a stable flat surface withstanding compressive stresses. Subsequently, all the undercuts in the access cavity walls were eliminated using a cylindrical-conical diamond bur with a 7-degree occlusal taper (Komet 846-018 M; Komet Den Komet Dental, Braseler GmbH & Co. KG, Lemgo, Germany), creating a continuous pulp chamber and access cavity. Additionally, all internal walls and axial line angles were smoothed and rounded. All the burs used during cavity design were mounted on a high-speed handpiece with water cooling. The dimensions of the resultant cavity were 7 mm ± 0.5 mm mesiodistally and 4 mm ± 0.5 mm buccolingually. The final standardized cavity design allowed the fabrication of an endo-crown restoration with an average of 2 mm thickness from the margins of the axial walls to the highest occlusal end of the restoration and an average of 4 mm thickness of the endo-core (extension of the endo-crown into the pulp chamber) (Fig. 1a).
- Conventional design with an extension in root canals (Group 2):
  This group involved the same procedures conducted in group 1 in addition to a 2 mm extension into the distal root canal, which was achieved after removing an additional 1 mm of root canal filling below the distal canal orifice. This was done using a Gate-Glidden drill #2 (Mani Inc., Utsunomiya, Tochigi, Japan), adapted on a low-speed handpiece without coolant to enhance visibility. To ensure equivalent intra-radicular extensions, the length of the Gate-Glidden drill was adjusted using a plastic silicone stopper at a fixed reference point (distal marginal ridge). Afterward, the root canal extension was irrigated with a saline solution to remove residual filling materials and dentin chips. Then, it was dried using oil-free, water-free air alongside a paper point ISO size 70 (Coltene Whaledent, Cuyahoga Falls, OH, USA). Finally, the continuity of the distal intra-radicular extension with the pulp chamber walls and the internal axial wall was checked (Fig. 1b).
- Conventional design with a buccal groove (Group 3):
  An axial groove, extending 1.5 mm into the center of the buccal wall, was created using a cylindrical-conical diamond bur with a 7-degree occlusal taper (Komet 846-018 M; Komet Dental, Braseler GmbH & Co. KG, Lemgo, Germany). This procedure was performed in conjunction with the conventional cavity design described in group 1. The groove measured 2.5 mm in length, extending from the occlusal limit of the buccal axial wall to the level of the pulp chamber roof. Importantly, it did not extend into the pulp chamber, ensuring adequate thickness of the peri-cervical dentine. This thickness is essential for the uniform dissipation of occlusal stresses along the root surface, which helps preserve the tooth’s fracture strength (Fig. 1c).

### Fabrication of endo-crown restorations

- Digital design (CAD):
  Following cavity preparation, a digital impression was captured using a high-resolution laboratory scanner (Dof Edge; Dof Inc., Seoul, South Korea) to ensure an accurate design for the restoration. This digital impression was then transferred to CAD (Computer-Aided Design) software (Exocad; Exocad GmbH, Darmstadt, Germany), where the restoration type was selected and specified as an “endo-crown.” The restoration was designed according to the previously mentioned dimensions, and a virtual try-in was performed by simulating the placement of the restoration digitally to ensure proper fit and adaptation.
- Digital fabrication (CAM):
  A lithium disilicate block (IPS e-max CAD; Ivoclar Vivadent AG, Schaan, Liechtenstein) was chosen with a medium-translucency and A2 shade. The CAD design was transferred to a wet milling machine (Ivoclar Vivadent, Schaan, Liechtenstein) to fabricate the endo-crown restorations. Then, the endo-crown restorations were allowed to dry before submission to crystallization firing in a ceramic furnace (Ivoclar Vivadent, Schaan, Liechtenstein) following the manufacturer’s guidelines to enhance translucency, aesthetics, and strength.
- Try-in:
  After fabrication, the restoration was seated on the respective tooth cavity to verify the proper margin adaptation. All margins were carefully investigated; after examination, all endo-crowns were found to be acceptable for cementation. Finally, restorations were finished and glazed prior to cementation.

### Cementation of endo-crown restorations

**Fig. 2:**
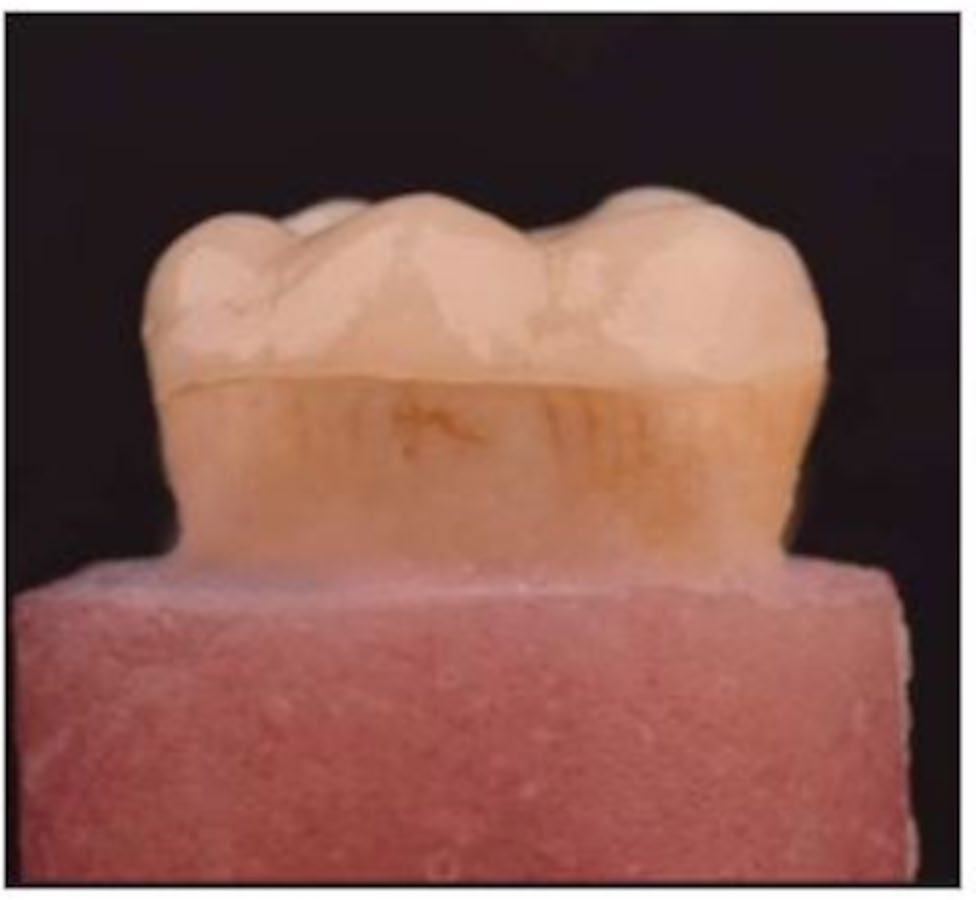
Representative image for the endo-crown after cementation

- Tooth surface treatment.
  For enamel etching, a 35% phosphoric acid etching gel (Ultra-etch, Ultradent, South Jordan, Utah, USA) was applied following the manufacturer’s instructions for 20 s. After etching, the area was rinsed with water for 20 s and dried using water-free and oil-free air for 5 s. Finally, the ED primer (Kuraray Noritake Dental, Kurashiki, Japan) was applied to the dentin, followed by gentle air drying for 5 s.
- Endo-crown surface treatment.
  According to the manufacturer’s recommendation, the intaglio surface of endo-crowns was etched using 9.5% hydrofluoric acid gel (Ultradent etch, Ultradent, South Jordan, Utah, USA) for 30 s, followed by water rinsing for 30 s. The etched surfaces were submitted to ultrasonic cleaning for 2 min to ensure the elimination of any dissolved debris and residual acid and air dried. Afterward, two thin coats of Silane (Ultradent Silane, South Jordan, Utah, USA) were applied per the manufacturer’s instructions using a micro brush for two intervals of 60 s each. Then, water-free and oil-free air was delicately sprayed to ensure uniform distribution and to remove the excess.
- Cementation process:
  Dual-cured resin cement (Dual CEM, Kiyomi Dental, Gijon, Spain) was mixed per the manufacturer’s instructions (auto-mix syringe mixing the base and catalyst in a 1:1 ratio). Following cement application on the intaglio surface of the endo-crown restoration and the entire cavity walls and floor (in addition to the root canal extension in group 2), the restoration was gently inserted and seated into the cavity under static finger pressure. In an attempt to standardize the cementation process, samples were positioned in a universal testing machine (Model 3345; Instron Industrial Products, Norwood, MA, USA). A consistent vertical static load of 10 N was applied for 5 min, followed by a brief light-curing for only 2 s. Finally, a spoon excavator was used to remove any excess cement. Subsequently, light curing was completed for 20 s on each tooth surface. Light curing was accomplished by a light-emitting diode curing unit (Blue phase, Ivoclar Vivadent, Zurich, Switzerland), with a light intensity of 1200 MW/cm² and wavelength bandwidth of 400–515 nm. To ensure adequate polymerization, the curing tip was in intimate contact with the tooth/restoration surface. The light output was standardized using the Aphrodite LED radiometer CM-2500 (Motion Medical Supplies & Equipment Corporation, Sanzhong, Taiwan) (Fig. 2).

Following cementation of the endo-crown restorations, all samples were kept at 37^o^C in distilled water for 24 h before being subjected to thermo-mechanical stress.

All restorative procedures were accomplished with a single experienced operator (M.G.A.).

Materials and instruments used throughout the current experiment with their technical specifications and manufacturers are listed in Table 1.

**Table 1:** The specification and manufacturer of the materials and instruments used in the study

Materials and instruments	Specification	Manufacturer
**IPS e-max CAD blocks**	Lithium disilicate glass-ceramic CAD/CAM materials available in a wide range of shades and translucencies to optimize aesthetic requirements, along with their mechanical and physical properties, which can be enhanced after crystallization.	Ivoclar Vivadent AG, Schaan, Liechtenstein
**Dual CEM**	- *A dual-cured resin cement. It is available in the following colors: A1*,* A2*,* A3*,* and TRANS in a 5 mg-syringe automixing the base and catalyst in a 1:1 ratio.* - *According to the manufacturer*,* this product is specifically designed for the permanent cementation of lithium disilicate*,* metal*,* and zirconia restorations.*	Kiyomi Dental, Gijon, Spain
**Ultra-etch**	35% phosphoric acid etching gel indicated for etching enamel and/or dentin surfaces	Ultradent, South Jordan, Utah, USA
**ED primer**	A self-etching primer employed for dentin bonding	Kuraray Noritake Dental, Kurashiki, Japan
**Ultradent etch and Silane**	Presented as two syringes, one contains a 9.5% hydrofluoric acid etchant gel for etching ceramic fitting surfaces, while the other contains silane to prepare the ceramic surfaces for bonding after etching.	Ultradent, South Jordan, Utah, USA
**Thymol solution**	- *Phenolic compounds have various applications in pharmaceutics*,* microbiology*,* and dentistry.* - *They exhibit strong antifungal*,* antiseptic*,* and preservative characteristics.* - *Standard concentration: 0.1%*	Formula e Acao, São Paulo, SP, Brazil.
**Elite HD+**	- *An addition-cured vinyl polysiloxane rubber base material used in dental impressions.* - *It features excellent detail reproduction*,* high dimensional stability*,* and hydrophilicity.*	Zhermack SpA, Badia Polesine, Italy
**Ethylenediaminetetraacetic acid (EDTA)**	- *A chelating agent available in gel and solution forms.* - *Gel form is used as a lubricant for mechanical instruments.* - *Solution form is used as a final rinse in conjunction with NaOCl to tackle its main limitation in removing the inorganic part of the smear layer.* - *pH: 7-8.5* - *Concentration: 10–17%* - *Solvent base: Aqueous or with surfactants for improved flow and penetration.*	Pulpdent, Watertown, MA, USA
**Gates-Glidden bur**	- *Rotary burs with a small flame-shaped head and a long shank designed for latch-type low-speed handpieces.* - *Material: Stainless steel.* - *Flutes: Non-cutting tip with radial flutes.* - *Sizes: # 1 to # 6* - *Applications: Coronal flaring*,* root canal filling removal*,* and post-space preparation*	Mani, Utsunomiya, Tochigi, Japan
**Hyflex CM files**	- *A multi-file engine-driven system used with torque-controlled endodontic motors in a continuous rotation motion.* - *Available in sizes of 25/0.08*,* 25/0.04*,* 20/0.06*,* 30/0.04 and 40/0.04*	Coltene Whaledent, Cuyahoga Falls, OH, USA
**Endo Z bur**	A tapered tungsten carbide bur with a non-cutting tip designed for refining endodontic access cavities while minimizing the risk of perforation.	Dentsply-Maillefer, Ballaigues, Switzerland

### Thermo-mechanical aging

Mechanical aging through cyclic loading was conducted using programmable logic-controlled equipment. This process utilized a newly developed four-station multimodal ROBOTA chewing simulator, which incorporates a thermo-cycling protocol and operates with a servo motor (Model ACH-09075DC-T, AD-TECH TECHNOLOG, Berlin, Germany). The ROBOTA chewing simulator features four chambers that simultaneously mimic vertical and horizontal movements under thermo-dynamic conditions. Each chamber includes an upper Jacob’s chuck, which serves as a hardened steel stylus antagonist holder that can be tightened with a screw, and a lower part made of Teflon that acts as the sample holder.

A weight of 5 kg, equivalent to a chewing force of approximately 49 N, was applied. The following settings were used for the chewing simulation: a cycle frequency of 1.6 Hz, a forward rising speed of 90 mm/s, a descending speed of 40 mm/s, a horizontal movement of 1 mm, and a vertical rising movement of 3 mm. To simulate six months of intraoral aging, the samples were subjected to 75,000 cycles, along with 5,000 thermal cycles (with temperatures alternating between 5˚C and 55˚C, and a dwell period of 25 s) [35, 36].

### Fracture resistance test (static fracture test)

**Fig. 3:**
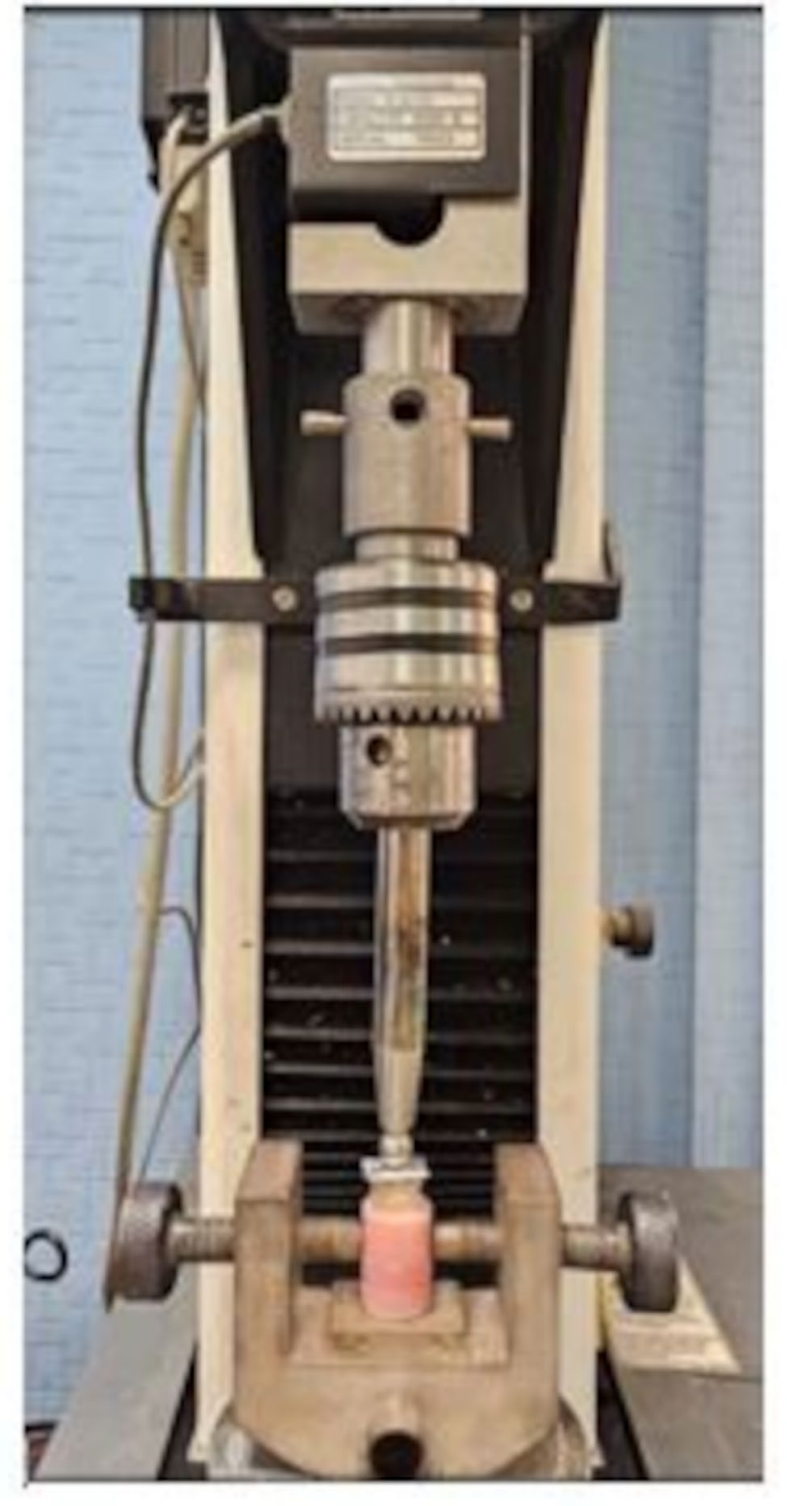
Representative image for a sample in the universal testing machine during fracture test

Each sample was independently mounted on a computer-controlled materials testing machine (Model 3345; Instron Industrial Products, Norwood, MA, USA) with a 5 kN load cell. Data were recorded using computer software (Bluehill Lite Software, Instron). Following the firm positioning of the sample in the lower compartment of the testing device with the tooth’s long axis at a 45° angle to the machine, the fracture test was done by compressive loading mode applied occlusally paralleling to the long axis of the roots using a metallic rod with a spherical tip (8.6 mm in diameter) attached to the upper movable compartment of the machine. The rod traveled at a cross-head speed of 1 mm/min with a tin foil sheet placed in between to achieve homogenous stress distribution and minimize the transmission of local force peaks. The load at failure was identified by an audible crack and confirmed by a sharp drop in the load-deflection curve as recorded using computer software (Bluehill Lite Software, Instron Instruments). The load required to cause fracture was recorded in Newton (Fig. 3).

### Failure mode analysis

An examiner blinded to the procedures (O.E.) assessed the failure modes using a USB Digital microscope with a built-in camera (U500x Digital Microscope, Guangdong, China) at a fixed magnification of 25×. Images were captured and transferred to a personal computer with image analysis software (Image J 1.43U, National Institute of Health, USA). Failure modes were categorized into five types [37];

- Type I: Repairable fracture (no visible fracture in either restoration or tooth structure),
- Type II: Repairable fracture (no visible fracture in the restoration; however, there is a fracture in the tooth structure),
- Type III: Repairable fractures (fractures limited to the restoration only),
- Type IV: Repairable fractures (fractures involve both the restoration and the tooth above the CEJ),
- Type V: Irreparable fractures (fractures involve both the restoration and the tooth and extend below the CEJ).

### Statistical analysis

Data were presented as the mean, standard deviation (SD), and range (Minimum– Maximum) for each value. The data distribution was examined using the Kolmogorov-Smirnov and Shapiro-Wilk tests to assess normality. A one-way ANOVA test was conducted to compare mean values, followed by Tukey’s post hoc test if a significant difference was observed. Additionally, a chi-square test was performed to compare failure modes. The sample size (*n* = 10 per group) was sufficient to detect large effect sizes for the main effects and pairwise comparisons, with a satisfactory power level set at 80% and a 95% confidence level. Results were analyzed using GraphPad Instat (GraphPad, Inc.) software for Windows. A *P* value of less than 0.05 was considered statistically significant.

## Results

The mean and standard deviation values of load to failure are presented in Table 2. Group 1 (conventional design) showed the highest statistically significant fracture resistance with the highest mean ± SD values of failure load (‎2521.2 ± 324.1‎ N) followed by group 3 (conventional design with a buccal groove extension) (‎1690.89 ± 129.72‎ N) and group 2 (conventional design with root canal extension) (‎1237.08 ± 201.81‎N) with statistically significant differences among the different study groups (*P* < 0.0001).

**Table 2:** The mean and standard deviation values (Mean ± SD) of fracture load values among the experimental groups

Variables		Mean ± SD	Range	95%CI	ANOVA
			Minimum -Maximum	Low - High	*P* value
**Experimental groups**	**Group 1**	2521.2 ± 324.1^A^	2197.1**-**2845.3	2320.32**-**2722.08	< 0.0001*
	**Group 2**	1237.08 ± 201.81^C^	1073.8**-**1640.7	1111.99**-**1362.16	
	**Group 3**	1690.89 ± 129.72^B^	1561.17**-**1820.6	1610.49**-**1771.28	

Different superscript letters indicating significant difference (Tukey’s *p* < 0.05) *; significant (*p* < 0.05), ns; non-significant (*p* > 0.05)

**Fig. 4:**
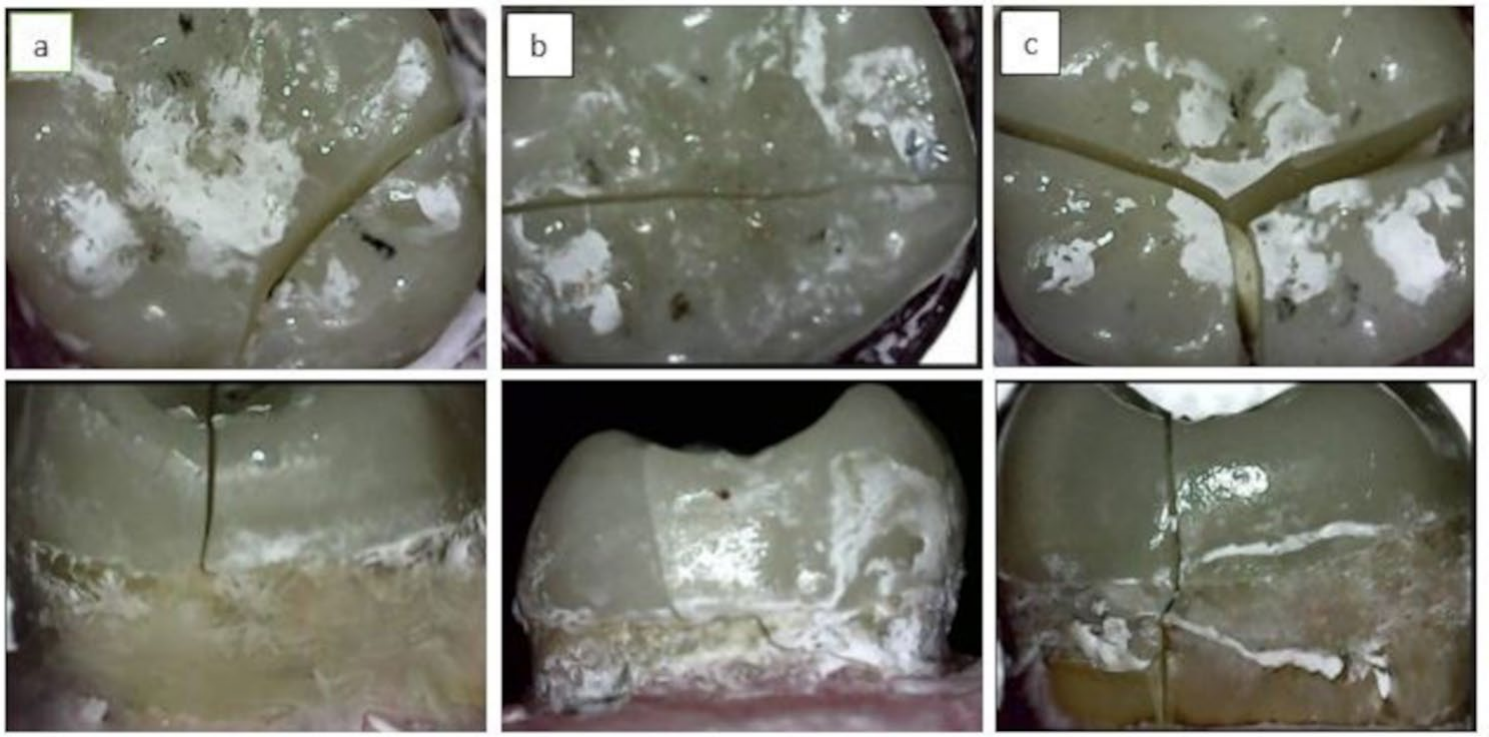
The various forms of failure modes from occlusal (above) and axial (below) views. **a**: Type III failure mode (fractures limited to the restoration only), **b**: Type IV failure mode (fractures involve both the restoration and the tooth above the CEJ), **c**: Type V: Irreparable fractures (fractures involve both the restoration and the tooth and extend below the CEJ)

**Fig. 5:**
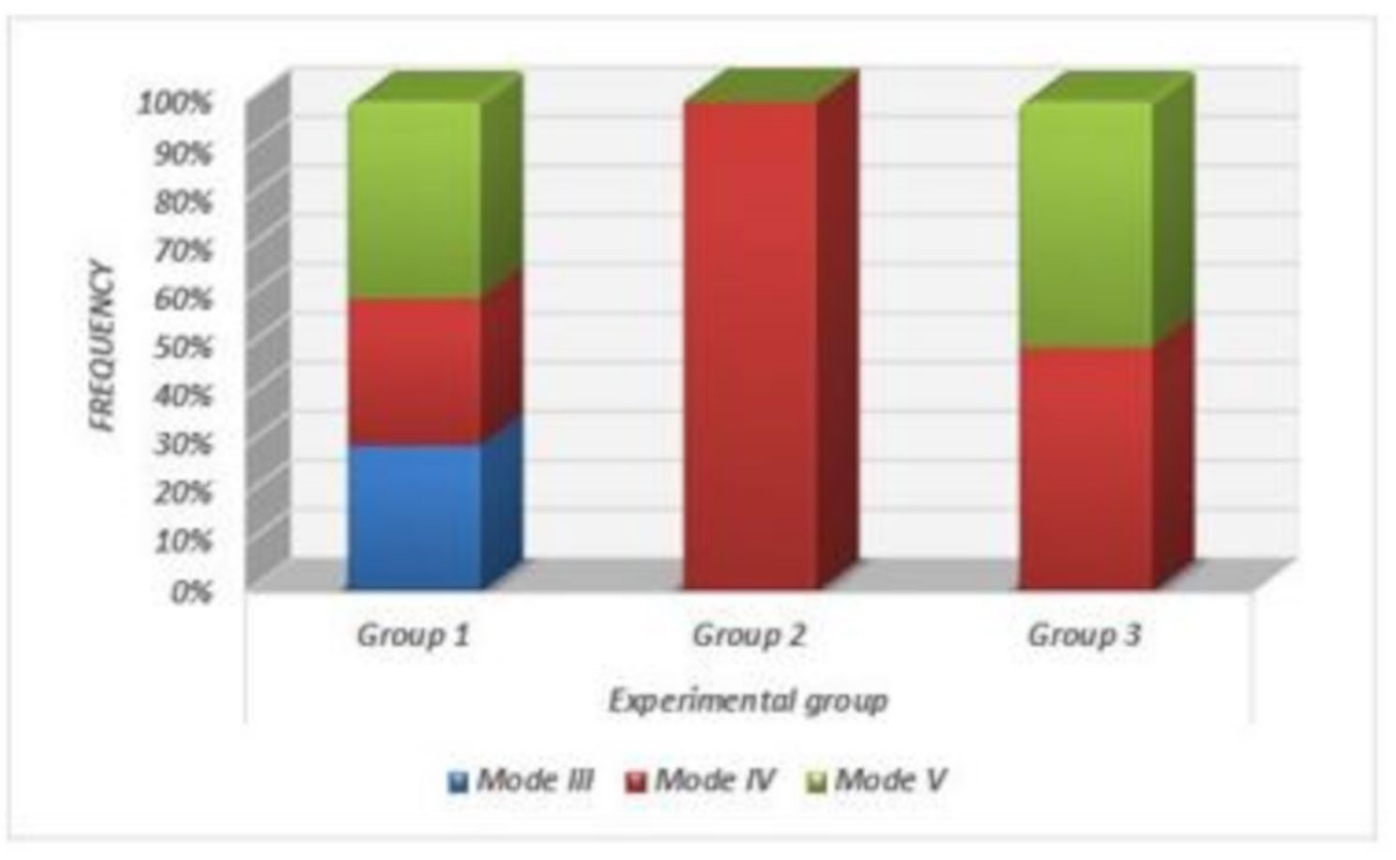
Bar chart showing failure mode distribution between tested groups

When comparing failure patterns across the different test groups, there were statistically significant differences among the three groups (*P* < 0.0001) (Figs. 4 and 5). Group 1 exhibited a higher occurrence of repairable failure patterns, with Modes III and IV each accounting for 30% of the samples, while 40% displayed an irreparable failure mode (Mode V). In Group 2, all samples demonstrated a Mode IV failure (100%), with no records of the other failure modes. Group 3 showed equal proportions of Modes IV and V, with each accounting for 50% of the samples, and no records of the other three failure modes. Notably, Type I and II failure patterns were not observed in any of the study groups.

## Discussion

Despite the decreasing trend of edentulism worldwide, challenges persist in maintaining oral health, with implications for dental care services. Restoring RCTT remains a pivotal aspect of dental practice, particularly due to the increased susceptibility of these teeth to fracture and biomechanical failure [38]. This increased risk is often attributed to the loss of tooth structure following root canal treatment, along with changes in the physical characteristics of the remaining dentin. Traditional restorative methods, such as the post-and-core technique with full coverage crowns, aim to retain the coronal restoration but often come with drawbacks like increased invasiveness and the risk of root perforation or fracture [12].

In contrast, endo-crown restorations, which utilize the pulp chamber for macro-mechanical retention and are bonded adhesively, present a more conservative and minimally invasive alternative [39]. This study was designed to evaluate the impact of the cavity design on the biomechanical performance of endo-crown restorations; conventional design, conventional design with root canal extension, and conventional design with buccal groove.

By investigating these designs, the study aimed to determine the most effective approach to restoring RCTT while preserving as much natural tooth structure as possible. Given the critical role of cavity design in stress distribution and retention [25, 26], the findings of this study provide valuable insights for optimizing the long-term success of endo-crown restorations, particularly for teeth with extensive coronal damage. Understanding the comparative biomechanical performance of these designs is essential for guiding clinicians in selecting the appropriate restorative strategy for RCTT.

The results demonstrated that the conventional design exhibited the highest fracture resistance, followed by the conventional design with buccal groove extension and the design with root canal extension, respectively, with statistically significant differences. As a consequence, the findings of the present study were able to reject the tested null hypothesis. These findings align with prior research and confirm the influence of cavity design on the mechanical behavior of endo-crown restorations [25, 27, 40].

The significantly higher fracture resistance in the conventional design supports previous studies that emphasized the importance of preserving the natural pulp chamber structure for optimal biomechanical performance [25, 26]. Additionally, the success of the conventional design mirrors reports by Gaintantzopoulou and El-Damanhoury [41], who highlighted that preserving as much tooth structure as possible allows better stress distribution and hence enhances fracture resistance.

Conversely, the lowest fracture resistance observed in the conventional design with root canal extension may be explained based on previous findings indicating that the intra-radicular extension can create more marginal and internal gaps, increasing stress concentration and reducing overall stability under load [41]. This result contrasts with previous research, which suggested that intra-radicular extensions might improve fracture resistance [27, 42]. On the other hand, prior findings concluded that the root canal extensions did not influence the fracture resistance of endo-crown restorations [43]. The differences may be due to variations in methodology, samples, materials, designs, bonding strategies, and fabrication techniques.

Axial grooves in cavity preparations have been proposed to enhance the retention and resistance forms of dental restorations by providing several advantages [44–46]: (1) They improve the mechanical interlocking between the restoration and the tooth structure, thereby increasing retention, (2) They help the restoration resist tipping or lateral forces and direct occlusal loads along the tooth’s long axis, which enhances stability (3) They promote more even stress distribution across both the restoration and the tooth, reducing the risk of debonding or fracture by preventing stress concentration at the margins, (4) They increase the surface area available for bonding, offering additional opportunities for micro-mechanical retention, (6) They enhance the stability of the restoration against rotational forces. Indeed, the conventional design with a buccal groove extension in the present study exhibited significantly superior fracture resistance when compared to the design with root canal extension. This result might be attributed to adding a groove that can improve the retention and stability of the restoration, allowing for better stress distribution under load. However, the groove design still did not match the performance of the conventional design, indicating that simpler designs may remain the most biomechanically stable option.

In an attempt to encompass all potential fracture scenarios, the present investigation included a more comprehensive classification of possible failure modes to draw the practitioner’s attention to every potential scenario. The failure mode analysis further supports the fracture resistance findings, with the conventional design and conventional design with buccal extension demonstrating a higher incidence of irreparable failures, while the intra-radicular extension design displayed more repairable failure patterns. This is consistent with a previous report that showed that stronger designs tend to fail under higher loads but with more severe consequences [47]. However, it is important to interpret the failure mode outcomes cautiously because the biomechanical behavior of the different endo-crown designs was tested under forces that exceed those typically encountered during normal oral functions [48]. Research indicates that occlusal forces in the molar area usually range from 100 to 200 N but can increase to as much as 965 N in cases of traumatic occlusal contact, parafunctional habits, or trauma [49]. In the present study, the average fracture values were above the normal and parafunctional forces in all groups. This can emphasize that the resultant fracture strength values are sufficient to resist occlusal forces in all three designs. It is worth mentioning that testing the fracture resistance is essential for assessing the survival of both restoration and root canal-treated teeth [26].

Due to their impact on the performance of endo-crown restorations, the present investigation has considered the endo-crown materials, method of fabrication, and adhesive cement [24]. Presently, various materials are available for fabricating endo-crown restorations, including resin composites, zirconia, zirconia-reinforced lithium silicate glass ceramics, and lithium disilicate glass ceramics [24]. Among these, lithium disilicate glass ceramics are highly favored due to their ability to bond effectively to tooth structure, favorable mechanical properties, and superior esthetics [50, 51]. Additionally, several studies have demonstrated that lithium disilicate glass ceramics possess the highest fracture resistance, even under lateral loading, when compared to other materials [50, 51]. Consequently, lithium disilicate was utilized in the present experiment.

There are two main approaches for fabricating endo-crown restorations: heat pressing or CAD/CAM. The CAD/CAM method holds many advantages as it can produce highly esthetic restorations with superior fit and quality in a single session [24]. That’s why the CAD/CAM technique was used in the current investigation. IPS e-max CAD blocks are a notable example of lithium disilicate glass ceramics that are compatible with the CAD/CAM method. This material provides a robust adhesive bond with resin cement and exhibits high flexural strength, ranging from 262 to 360 MPa, along with a fracture toughness between 2.0 and 2.5 MPa after crystallization, ensuring its durability [52]. In addition, IPS e-max CAD boasts excellent aesthetic characteristics, offering a wide range of translucencies and shades [52]. In the present study, IPS e-max CAD with medium translucency and A2 shade was selected. The medium translucency strikes a balance between translucency and the ability to mask any discolored underlying tooth structure, making it suitable for use in both anterior and posterior regions. A2 color was chosen as it is the most commonly used shade in dental restorations.

Adhesive cement is essential for the performance and durability of endo-crown restorations, as it provides micromechanical retention [24]. Effective adhesion helps distribute stress across the restoration, enhancing its fracture resistance [15]. Resin cements are commonly used for cementing endo-crown restorations due to their excellent bonding capabilities, low solubility, and favorable aesthetic and mechanical properties [51]. Based on their polymerization methods, they can be categorized into self-cured, light-cured, and dual-cured. Self-cured resin cements are rarely utilized because they have shorter working times and inferior aesthetic and mechanical properties [24]. On the other hand, light-cured resin cements have a longer working time, but their effectiveness may be limited in deep cavities where light cannot penetrate. Therefore, they are typically recommended only for shallow preparations [24]. Dual-cured resin cements combine the advantages of both self-cured and light-cured resin cements and can be used in deep cavities [24]. Consequently, dual-cured resin cement was employed in this study.

Endo-crown restorations require different preparation criteria than traditional crowns, primarily due to their reliance on anchorage to the pulp chamber and the cavity margins to establish macro-mechanical retention [53]. For composite materials, an occlusal reduction of 1 to 1.5 mm is recommended due to their elastic properties and ability to absorb stresses [54]. In contrast, a minimum occlusal reduction of 2 mm is suggested for ceramic materials [54]. When comparing the preparation designs, the butt joint design is simpler and offers better adaptation and marginal integrity than the shoulder design with a ferrule. Some modifications in the preparation can be performed in order to fulfill biomechanical, aesthetic, or various material requirements [53]. The main innovation presented in this investigation is the option of utilizing a buccal groove instead of relying on intra-radicular extensions when additional retention and resistance are necessary. This approach is particularly relevant for severely damaged teeth with shallow pulp chambers, weak axial walls, or those subjected to excessive functional or parafunctional forces, which may contraindicate the endo-crown restoration with the conventional design. It emphasizes the concept of minimally invasive techniques, which could potentially improve fracture resistance.

The dental equator (DE) refers to the widest circumference of a tooth and is pivotal in managing the distribution of stress at the interface between the tooth structure and restoration materials. When assessing the fracture resistance of indirect restorations, such as endo-crowns, the precise positioning of the restoration margin in relation to the DE emerges as a crucial factor [55]. Supragingival margins, which are placed above the dental equator, offer several advantages. They can streamline clinical procedures by improving isolation due to reduced moisture interference, increasing accessibility, and facilitating a more efficient finishing process. Nevertheless, this strategic placement may come at the expense of the mechanical resistance of the restoration, as the lack of subgingival support could lead to compromised integrity under functional loads. In contrast, subgingival margins that are located below the DE can significantly benefit the restoration’s structural integrity. This placement provides enhanced axial wall support and improves the resistance form of the restoration, which is critical in preventing fracture under occlusal forces. These factors could lead to a higher overall fracture resistance of the endo-crown. Nonetheless, the advantages of subgingival margins must be carefully weighed against the difficulties they pose, including challenges in moisture control and ensuring optimal bonding in these sub-equatorial areas where saliva and blood contamination may be more prevalent. While the current study standardized margin location to isolate the impact of cavity design on restoration performance, the implications of margin positioning for the long-term success of endo-crown restorations necessitate further exploration. Future research should thoroughly investigate these dynamics, considering not only fracture resistance but also the overall efficacy and longevity of such restorative techniques in varying clinical scenarios.

To minimize the risk of bias and ensure valid outcomes, this study placed significant emphasis on selecting samples based on the reasons for extraction, the age, and the systemic condition of the patients. Being the most common teeth requiring root canal treatment, intact mandibular first molars were collected from healthy adult patients within a similar age range to reduce the likelihood of any pathological or physiological changes in dentin. This approach helps ensure that the volumes of the pulp chambers are comparable, which can influence the biomechanical performance of endo-crowns [48]. In addition, it has been reported that age significantly impacts the bond strength of restorations to dentin [56]. The study also accounted for other factors that could adversely affect the bond to dentin, including extraction time and technique, storage duration, and the media used [31].

There are two main approaches for testing fracture resistance: dynamic and static fracture tests. While the dynamic fracture test closely resembles clinical situations, its variations can lead to difficulties in achieving reproducible results. As a result, the static test has become more popular due to its reliability and effectiveness [57] and thus was applied in the present investigation.

This investigation has certain limitations that should be considered when interpreting the outcomes, including being conducted under controlled conditions and not considering patient-specific factors. It also did not consider variations in endo-crown materials, cements, bonding strategies, and different methods that can be used for fabrication and testing, which may not reflect the variability in clinical scenarios. Different materials and techniques could yield different results, highlighting the need for further investigations considering such differences. Moreover, the present study employed a class I cavity, which is easier to standardize than the other cavity types, allowing better comparison of the test groups. However, this is not the case in most clinical situations, as root canal-treated teeth often are mutilated with significant tissue loss. Furthermore, in the present study, standardized cavity depths of 4 mm were implemented to allow for better comparison of both designs, comprising extensions, to the conventional design. However, this approach overlooks a wide range of complex clinical scenarios, including shallow cavities, where stability and retention are critically challenging [24]. Consequently, future research comparing these designs in teeth with shallow cavity depths is essential to provide a holistic grasp of the influence of cavity designs on the clinical performance and longevity of endo-crown restorations in diverse clinical conditions. These studies may necessitate the use of different restorative materials and cement protocols as the interplay between the cavity preparation, the type of endo-crown restorative materials, and the cement system is critical in determining the overall success and longevity of the endo-crown restorations [58].

Different cavity depths can affect the robustness of mechanical interlocking, bonding quality, and degree of load distribution. Deep cavity preparations (4–5 mm) typically improve the retention and stability of rigid restorative materials like lithium disilicate ceramics, which possess a high modulus of elasticity that requires a large surface area for effective distribution of occlusal forces [59]. Nevertheless, increasing the cavity depth may adversely impact the marginal adaptation of endo-crown restorations due to bonding concerns [26]. To address this issue, dual-cured resin cement is highly recommended in such deep cavities to ensure complete polymerization and promote a strong chemical bonding to these silica-based ceramics, thereby enhancing marginal adaptation and retention [24]. Zirconia-based ceramics is another type of rigid ceramic material. Despite their high strength, their rigidity, which surpasses that of lithium disilicate ceramics, can lead to catastrophic failures, alongside their inferior retention requires meticulous consideration during cavity preparation and cementation [60, 61]. Like lithium disilicate materials, to tackle their extreme stiffness, zirconia-based ceramics require increasing the cavity depths, which may compromise adequate bonding in deeper areas, jeopardizing retention [61]. Thus, specific cementation protocols, including the use of dual-cured resin and appropriate surface treatments, along with deep preparations, have the potential to improve both chemical and mechanical retention of such rigid restorations. For rigid ceramics, considering the elastic modulus of the luting cement is paramount since it can influence stress distribution [58]. Cements with an intermediate modulus of elasticity are capable of buffering stresses between rigid ceramics and tooth structure, minimizing interfacial failure [58]. Additionally, surface treatments play a vital role in enhancing cement adhesion and retention, irrespective of the cement type [61]. With increasing cavity depth for such rigid ceramic materials, cement space is an important factor to be considered. A prior study demonstrated that the cement space can affect the quality of marginal adaptation [62].

Conversely, hybrid ceramics, polymer infiltrated ceramic network, resin composite, and PEEK, which exhibit a modulus of elasticity closer to that of dentin structure, offer adequate marginal adaptation and retention with more reparable fractures even in shallow cavities (2–3 mm), making their use in teeth with limited tooth structure is suitable [47, 59]. Therefore, they can benefit from simplified cementation protocols like self-adhesive or light-cured resin cements.

Moreover, the design of the cavity margin is a critical factor for successful endo-crown restorations. Research highlighted that margin design can affect the selection of restorative material and cement to ensure better stress distribution [63].

As such, it is imperative for clinicians and researchers alike to consider all these factors, tailoring the most appropriate cavity design, endo-crown restorative material, and cementation protocol according to each individual situation to optimize the clinical outcomes of endo-crown restorations.

Since the major limitation of the current research lies in its ex-vivo design, therefore, prospective large-scale randomized clinical trials are required to bolster the validity and reliability of the present findings. Despite the ex-vivo design, the experimental procedures in the present study were closely aligned with the real clinical practice. Teeth roots were invested in a silicone material encased by acrylic resin extending 2 mm apically to CEJ. This procedure was intended to replicate the periodontal attachment and bone support, respectively. It has been reported that root embedment is a critical step that can affect the validity of the fracture resistance results [64]. In addition, all root canal procedures, including access cavity preparation, root canal preparation, and root canal filling, were performed similarly to the clinical setting. Unlike numerous laboratory investigations that employed saline or distilled water to exclude the adverse NaOCl impact on the physical and mechanical characteristics of dentin, 2.5% NaOCl was used in the current study to mimic the clinical procedures [65]. To simulate the natural stresses in the oral environment, samples in the current study were submitted to aging procedures.

Given these methodological strengths, the present study has the potential to provide significant clinical implications.

## Conclusion

The study’s findings bring forth significant insights into the biomechanical performance of endo-crown restorations in different cavity designs. From the current study, the following conclusions can be drawn:

1. The conventional design demonstrated the highest fracture resistance, emphasizing the importance of preserving the natural pulp chamber structure to maintain optimal mechanical stability.
2. When further extensions are needed, the buccal groove design is preferred over the root canal extension design.

Overall, the study highlights the biomechanical advantages of simpler, conventional endo-crown designs in restoring root canal-treated teeth, making them a more reliable option for clinical application. These results can guide clinicians in selecting the most effective and conservative restorative approach to ensure long-term success.

## Data Availability

Availability of the data and materials: All data or materials generated or analyzed during this study are included in this article.

## Declarations

### Clinical trial number

Non-applicable. Conducting the current experiment was limited to the approval of the local Research Ethics Committee at the Faculty of Dentistry, Minia University, Egypt (committee no. 94, registration no. 705, Date: 28/2/2023).
